# EPMLR: sequence-based linear B-cell epitope prediction method using multiple linear regression

**DOI:** 10.1186/s12859-014-0414-y

**Published:** 2014-12-19

**Authors:** Yao Lian, Meng Ge, Xian-Ming Pan

**Affiliations:** The Key Laboratory of Bioinformatics, Ministry of Education, School of Life Sciences, Tsinghua University, Beijing, 100084 China; CAS Key Laboratory of Genome Sciences and Information, Beijing Institute of Genomics, Chinese Academy of Sciences, Beijing, China

**Keywords:** B-cell, Linear epitope, Prediction, Multiple linear regression

## Abstract

**Background:**

B-cell epitopes have been studied extensively due to their immunological applications, such as peptide-based vaccine development, antibody production, and disease diagnosis and therapy. Despite several decades of research, the accurate prediction of linear B-cell epitopes has remained a challenging task.

**Results:**

In this work, based on the antigen’s primary sequence information, a novel linear B-cell epitope prediction model was developed using the multiple linear regression (MLR). A 10-fold cross-validation test on a large non-redundant dataset was performed to evaluate the performance of our model. To alleviate the problem caused by the noise of negative dataset, 300 experiments utilizing 300 sub-datasets were performed. We achieved overall sensitivity of 81.8%, precision of 64.1% and area under the receiver operating characteristic curve (AUC) of 0.728.

**Conclusions:**

We have presented a reliable method for the identification of linear B cell epitope using antigen’s primary sequence information. Moreover, a web server EPMLR has been developed for linear B-cell epitope prediction: http://www.bioinfo.tsinghua.edu.cn/epitope/EPMLR/.

**Electronic supplementary material:**

The online version of this article (doi:10.1186/s12859-014-0414-y) contains supplementary material, which is available to authorized users.

## Background

The humoral immune response is based on the amazing ability of antibodies to recognize and bind to antigens of intruding organisms, such as bacteria and viruses [1]. Antibodies bind specifically to a contiguous amino acid sequence of a protein known as the linear B-cell epitope or to a folded structure formed by discontinuous amino acids known as the conformational B-cell epitope [2,3]. Prediction of B-cell epitopes is critical for immunological applications. Specifically, predicted peptides can be synthesized and can be used to replace the intact antigen molecules as reagents for detecting anti-protein antibodies in immunoassay [4], as immunogens for raising anti-peptide antibodies to cross-react with the protein of interest [5], or in the development of synthetic peptide vaccines [6]. Although the majority of B-cell epitopes are conformational [7], most B-cell epitopes prediction approaches concentrate on the “easier” linear epitopes [8].

Earliest linear B cell epitope prediction models were based on propensity profiling. Blythe and Flower [9] demonstrated that the propensity profiling methods cannot be used to reliably predict the epitope. Even the best propensity profiling method only yielded a success rate marginally better than that produced randomly using a receiver operating characteristics (ROC) plot. Later, machine learning methods have been explored to improve the prediction performance [10-22]. However, most of these methods were developed on very small datasets (~872 epitopes and non-epitopes) with negative dataset that were randomly selected peptides instead of experimentally verified non-epitopes [23].

In this work, based on the antigen’s primary sequence information, a novel linear B-cell epitope prediction model was developed using the multiple linear regression (MLR). A large dataset called BEOD which was derived from BEOracle dataset [19] was used to train and test our model. It is worthwhile to note that all epitopes and non-epitopes of our BEOD dataset were experimentally verified. Nevertheless, experimental non-epitope data still have the potential to be epitopes due to flawed interpretation of the results or simple experimental errors [24]. Models built on different subsets of such noisy negative dataset may produce very different results. In order to alleviate the noisy problem caused by the negative dataset and report a reliable prediction result of our model, we have performed 300 experiments utilizing 300 sub-datasets of which each negative sub-dataset was randomly selected from the BEOD negative dataset while each positive sub-dataset was the unchanged BEOD positive dataset. 10-fold cross-validation was employed to evaluate the performance of our model. Our model produced average sensitivity (Sn) of 81.8%, precision (P) of 64.1% and area under the receiver operating characteristic curve (AUC) of 0.728 over the 300 experiments. A web server EPMLR implementing linear B cell epitope prediction is available at: http://www.bioinfo.tsinghua.edu.cn/epitope/EPMLR/.

## Results

### Sliding window size selection

**Figure 1:**
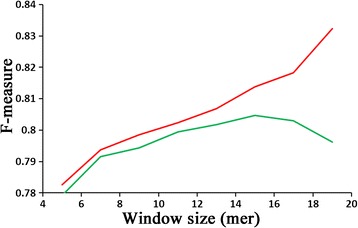
**The effect of the sliding window size on the overall prediction F-measure.** Red: Self-consistency test; Green: 10-fold cross-validation test.

To evaluate the effect of sliding window size n on the prediction performance, we conducted modelling trials on BEOD dataset using different window sizes from 5 to 19, representing the range in which peptides can be synthesized relatively easily for immune experiments. As shown in the Figure 1, the F-measure value of 10-fold cross-validation test achieved its highest value when the window size n was 15. Moreover, at 15 point, the F-measures obtained by the 10-fold cross-validation test and the self-consistency test are very close to each other, which further validates the reliability of the performance using sliding window size of 15. It is generally accepted that the closer the F-measures obtained by the cross-validation and self-consistency tests are, the more reliable the performance of the cross-validation test is. Therefore, in this work, 15 was set as the default window size.

### Prediction performance

**Figure 2:**
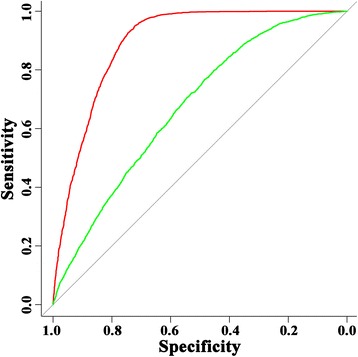
**ROC curves of the best and worst performance among 300 modeling trials using 10-fold cross-validation.** Red: the best performance; Green: the worst performance.

We performed 300 experiments on 10-fold cross-validation utilizing 300 sub-datasets that are the same in the positive datasets but different in the negative datasets. For each trial, the positive dataset of 4405 epitopes are exactly same with BEOD’s 4405 epitopes while the negative dataset of 4405 non-epitopes are randomly selected from BEOD’s 8467 non-epitopes. The ROC plots for the best and worst performances among the 300 trials are shown in Figure 2. The performances of all 300 trials are summarized in Table 1. As shown in Table 1 and Figure 2, the variance of the 300 results is large, with Sn ranging from 83.5% to 81.7%, P from 77.6% to 55.7%, F-measure from 0.805 to 0.663, and AUC from 0.893 to 0.673. These large discrepancies corroborate our speculation of the noise of non-epitopes even if they are experimentally verified and support our means of randomly constructing many negative sub-datasets and reporting the average result instead of the best result. In conclusion, our sequence-based linear B-cell epitope prediction method achieved an average Sn of 81.8 ± 0.8% (95% CI), P of 64.1 ± 0.2% (95% CI), F-measure of 0.719 ± 0.08 (95% CI), and AUC of 0.728 using 10-fold cross-validation.

**Table 1:** **Summary of the 300 trials’ performances using 10-fold cross-validation**

**Performance**	**Sn (%)**	**P (%)**	**F-measure**	**AUC**
Best	83.5	77.6	0.805	0.893
Worst	81.7	55.7	0.663	0.673
Average	81.8 ± 0.8	64.1 ± 0.2	0.719 ± 0.08	0.728

### Comparison with Other Prediction Methods

**Figure 3:**
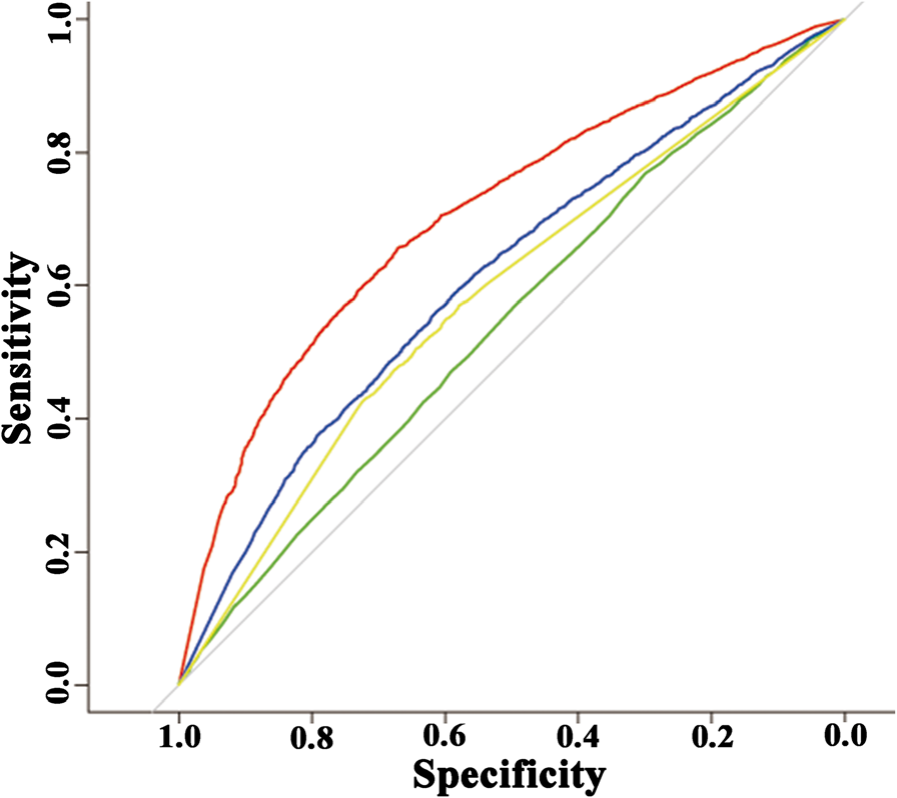
**ROC curves of ABCpred, BCPred, AAP and our EPMLR method performed on the BEOD dataset.** Green: ABCpred; Blue: BCPred; Yellow: AAP; Red: EPMLR.

We compared our EPMLR method with the methods of ABCpred [10], AAP [11] and BCPred [13] through applying their web servers to the BEOD dataset. The ROC plots for performances of ABCpred, AAP, BCPred and EPMLR are shown in Figure 3. The AUC values for ABCpred, AAP, BCPred and EPMLR are 0.547, 0.582, 0.615 and 0.728, respectively. It is clear from the ROC plots that EPMLR produced better performance in comparison with ABCpred, AAP and BCPred.

Next, we compared our method with SVMTriP method which is a recently published large dataset based method [21]. We performed a 5-fold cross-validation on the SVMTriP dataset. Our method obtained Sn of 80.56% and P of 54.9% which is similar to the performance of SVMTriP method (Sn of 80.1%, P of 55.2%) using 5-fold cross-validation. Our method observed similar Sn (81.8% vs. 80.56%) but a decreased P (64.1% vs. 54.9%) on the BEOD dataset and SVMTriP dataset. The decreased P value could be resulted from the fact that the negative non-epitope dataset of the SVMTriP dataset was from the remaining segments which have not been marked as epitopes in the corresponding antigen sequences.

Similarly, we compared with LBtope method which is the most recently published large dataset developed method [25]. We applied our method to the Lbtope_Fixed_non_redundant dataset (LFNR) whose epitopes and non-epitopes were all experimentally verified. Using the same experimental procedure of LBtope, on the LFNR dataset, our method obtained an AUC of 0.62, which is comparable to the AUCs (0.57 ~ 0.69) obtained by LBtope method by training using 5-fold cross-validation on 90% of the data and testing on the remaining 10% of the data with various features.

Table 2 lists the comparison of our EPMLR with these methods in detail.

**Table 2:** **Comparison of EPMLR with other methods**

**Methods**	**Dataset used**	**Performances**				
		**Sn (%)**	**Sp (%)**	**P (%)**	**Acc (%)**	**AUC**
ABCpred	BEOD	57.19	51.75	54.24	54.47	0.547
AAP		60.25	54.01	40.53	56.15	0.582
BCPred		65.04	51.87	41.28	56.38	0.615
EPMLR		81.79	45.87	64.07	63.83	0.728
SVMTriP	SVMTriP	80.1	Unavailable	55.2	Unavailable	0.702
EPMLR		80.56	32.30	54.9	56.43	0.644
LBtope	LFNR	54.38 ~ 65.88	57.31 ~ 63.97	Unavailable	55.85 ~ 64.86	0.57 ~ 0.69
EPMLR		60.76	56.14	57.99	58.45	0.62

## Discussion

The development of epitope prediction research was accompanied by the development of a large and experimentally well-characterized dataset that comprises both positive epitopes and negative non-epitopes [26]. In contrast to the simplicity of the construction of a positive dataset, the construction of a negative dataset has been still debated. Non-epitopes were not used in the early studies. Some authors attempted to construct negative datasets by randomly choosing peptides either from a protein database (such as Swiss-Prot) where no antibody binding is reported or from the antigen areas not encompassing any of the reported epitopes. In recent years, researchers have begun to construct negative datasets from the immune epitope database IEDB [27] database. IEDB collects both epitopes and non-epitopes from experimentally validated data. However, experimental non-epitope data still have the potential to be epitopes due to flawed interpretation of the results or simple experimental errors [24]. Thus, models built on different subsets of such uncertain dataset may produce uncertain predictions, as demonstrated by the results of the 300 trials of our model. Although we can produce a good result by subjectively selecting a self-reinforcing negative dataset, the reliability of such good performance is not guaranteed. Thus, in this work, we performed many parallel trials using the same positive dataset but different negative datasets that are randomly selected from the noisy negative dataset and reported the average of all results as the final result. Such an averaging method could help produce a reliable result.

## Conclusions

In this work, a novel sequence-based linear B cell epitope prediction model was developed. A web server EPMLR implementing the prediction is available at: http://www.bioinfo.tsinghua.edu.cn/epitope/EPMLR/. As a reliable method developed based on a large dataset, EPMLR offers new insights into the linear B cell epitope prediction and a new option for scientists to do their prediction.

## Methods

### Datasets

In this work, we used BEOracle dataset because it is a large dataset and both epitopes and non-epitopes of BEOracle dataset were experimentally verified. Through combining entries from IEDB, BCIPEP and AntiJen databases, Wang and his colleagues constructed the BEOracle dataset [20]. They extended these epitope sequences equally on both sides to get epitopes of a final length of 100 amino acids using the Uniprot identifiers associated with them. Further, we trimmed BEOracle dataset (100-mer) from both ends equally to extract the core 20-mer peptides. Finally, we obtained 4,405 epitopes and 8,467 non-epitopes and we called this unbalanced BEOracle-Derived dataset (BEOD).

To alleviate the problem caused by the noise of negative dataset, we then constructed 300 sub-datasets which were the same in the positive dataset but different in the negative dataset. Each of the 300 sub-datasets contained the whole 4,405 epitopes of BEOD and an equal number of 4,405 non-epitopes randomly selected from the 8,467 BEOD non-epitopes. These 300 sub-datasets were used to perform the 300 experiments using the same algorithm.

The SVMTriP dataset, which was introduced by Yao B *et al.* [21], consists of 4925 epitopes and 4925 non-epitopes. Originally, total of 65,456 B-cell linear epitopes were downloaded from IEDB (version June 11th, 2012) and the identical epitopes and those possibly related to T-cell are removed. Next, truncation and extension technique was applied to get fixed length pattern. Finally, 4925 non-redundant epitope sequences were obtained after > = 30% similarity process by BLAST [28]. For the negative dataset, the same number of equal-length sub-sequences were extracted from the non-epitopic segments in the corresponding antigen sequences.

The Lbtope_Fixed_non_redundant dataset (LFNR), which was introduced by Singh H *et al.* [25], consists of 7824 B-cell epitopes and 7853 non-epitopes. Originally, total of experimentally validated 49694 B-cell epitopes and 50324 non B-cell epitopes were obtained from the IEDB in Jan 2012. After truncation and extension, sequences with fixed length were created. Then identical epitopes and common patterns in both types of patterns were removed. Finally, after 80% non-redundant process by CD-HIT [29], 7824 B-cell epitopes and 7853 non-epitopes were kept. This non-redundant and fixed length dataset was named Lbtope_Fixed_non_redundant.

### Algorithm

In this study, we constructed an epitope prediction model based on primary sequence information. The modeling trial was performed as follows.

Each 20-mer epitope (or non-epitope) was scanned step by step using a sliding window of *n* residues. We use *ω*_*i*_ to represent the epitope state of a window *A*_*i*_: if a window is from an epitope input, its *ω*_*i*_ is epitope, otherwise is non-epitope. Defining *I*(*ω*_*i*_) as the epitope indicator of the window, the value of *I*(*ω*_*i*_) is taken as 1 when the window is being in the state of epitope, otherwise as 0. We assumed that *I*(*ω*_*i*_) is a function of the linear combination of features derived from the sequence and physical-chemical properties of the window. Therefore, for a window we have equation (1):

1 $$ \begin{array}{l}I\left({\omega}_i\right)={\displaystyle \sum_{j=1}^n\alpha \left(1,2\dots 19\Big|\omega \right){R}_j}+{\displaystyle \sum_{j=1}^{n-1}}{\displaystyle \sum_{k=j+1}^n}{\beta}_{j,k}\left(\omega \right){B}_j{B}_k\\ {}\kern4.5em +{\displaystyle \sum_{j=1}^{n-1}}{\displaystyle \sum_{k=j+1}^n}{\gamma}_{j,k}\left(\omega \right){S}_j{S}_k+{\displaystyle \sum_{j=1}^{n-1}}{\displaystyle \sum_{k=j+1}^n}{\delta}_{j,k}\left(\omega \right)V\left({R}_j{R}_k\right)+C\left(\omega \right)\end{array} $$

Here, subscripts *j* and *k* denote position *j* and *k* in the window. *R*_*j*_ is a 19-D vector with the component for the residue at position *j* as 1 and the others as 0. *α*(1, 2 … 19|*ω*) is the coefficient vector for 19 amino acids (with one omitted). ${\displaystyle \sum_{j=1}^n\alpha \left(1,2\dots 19\Big|\omega \right){R}_j}$ represent the features of occurrence of amino acid type from the first position to the last position for an n-mer window sequence. *B*_*j*_ and *B*_*k*_ are the normalized hydrophilicity values of residues at positions *j* and *k*, while *β*_*j*,*k*_(*ω*) is the coefficient combining the residue pair. ${\displaystyle \sum_{j=1}^{n-1}}{\displaystyle \sum_{k=j+1}^n}{\beta}_{j,k}\left(\omega \right){B}_j{B}_k$ represent the features of autocorrelation of the hydrophobicity index of residue pair (residue *R*_*j*_ at position j and residue *R*_*k*_ at position k) for an n-mer window sequence. Similarly, *S*_*j*_ and *S*_*k*_ are the normalized side chain mass values of residues at positions *j* and *k*, while *γ*_*j*,*k*_(*ω*) is the coefficient combining the residue pair. ${\displaystyle \sum_{j=1}^{n-1}}{\displaystyle \sum_{k=j+1}^n}{\gamma}_{j,k}\left(\omega \right){S}_j{S}_k$ represent the features of autocorrelation of the side chain mass of residue pair for an n-mer window sequence. *V*(*R*_*j*_*R*_*k*_) is a 500-D vector whose components refer to 500 most important position specific residue pairs *R*_*j*_*R*_*k*_, while *δ*_*j*,*k*_(*ω*) is the coefficient combining the residue pair. In model training, we compared the 500 *R*_*j*_*R*_*k*_ with all *R*_*j*_*R*_*k*_ (*n* × (*n* − 1)/2 in total) existed in a window, the value of a component of *V*(*R*_*j*_*R*_*k*_) is set as 1 if the *R*_*j*_*R*_*k*_ to which the component referred exists in the window, otherwise as 0. ${\displaystyle \sum_{j=1}^{n-1}}{\displaystyle \sum_{k=j+1}^n}{\delta}_{j,k}\left(\omega \right)V\left({R}_j{R}_k\right)$ represent the feature of occurrence of selected residue pairs in an n-mer window sequence.

The 500 residue pairs were selected according to the following procedures: we firstly calculated the occurrence frequency for all *R*_*j*_*R*_*k*_ (20 × 20 × *n* × (*n* − 1)/2 in total) in the training dataset and eliminated *R*_*j*_*R*_*k*_ with occurrence frequency less than the average value (about 50%) for statistic stability. We then calculated the information value *D*(*R*_*j*_*R*_*k*_) of the remaining *R*_*j*_*R*_*k*_. *D*(*R*_*j*_*R*_*k*_) is defined as equation (2):

2 $$ D\left({R}_j{R}_k\right)={\displaystyle \sum_{t=0}^1{f}_t}\left({R}_j{R}_k\right)\times \log \left(\frac{f_t\left({R}_j{R}_k\right)}{P_t}\right) $$

where *f*_*t*_(*R*_*j*_*R*_*k*_) represents the occurrence frequency of a *R*_*j*_*R*_*k*_ derived from the epitope (*t* = 1) and non-epitope (*t* = 0) in the training dataset, respectively. *P*_*t*_ represents the naturally occurring probability of a *R*_*j*_*R*_*k*_ based on the relative sizes of the epitope and non-epitope datasets in the training dataset (for example, *P*_1_ = *P*_2_ = 0.5 if the size of the epitope dataset is equal to the size of the non-epitope dataset). All *D*(*R*_*j*_*R*_*k*_) values were ranked by the descending orders. Finally, 500 *R*_*j*_*R*_*k*_ with the largest value of *D*(*R*_*j*_*R*_*k*_) were selected. Here, we selected 500 components because the curve of all *D*(*R*_*j*_*R*_*k*_) values by descending order shows as exponential decay and the point of inflection is about 500 ( Additional file 1).

On the training dataset, all the fitting coefficients in Equation (1) were determined by the MLR method [30]. Once the coefficient matrix is obtained, we adopted the same sliding window procedure with the 20-mer peptides on the testing dataset. Each of the *n*-sized window *ω*_*i*_ of the 20-mer peptide was predicted to be an epitope or not with an epitope propensity score *Q*(*ω*_*i*_). For any 20-mer peptide, there are 21 − *n* windows and the epitope propensity score of the 20-mer peptide was calculated by taking the average of all 21 − *n Q*(*ω*_*i*_) scores. In this representation, every 20-mer peptide in the testing dataset is scored for its propensity to be an epitope or a non-epitope.

### Performance Measures

In 10-fold cross-validation test, the original dataset is randomly partitioned into 10 equal size subsets. Of the 10 subsets, a single subset is retained as the validation data for testing the model, and the remaining 10-1 subsets are used as training data. The cross-validation process is then repeated 10 times, with each of the 10 subsets used exactly once as the validation data. The 10 results can then be averaged to produce a single estimation.

Sn, P, F-measure and accuracy (Acc), are defined in the following equations:

$$ {S}_n=\frac{TP}{TP+FN}\times 100\% $$

$$ P=\frac{TP}{TP+FP}\times 100\% $$

$$ \mathrm{F}=\frac{2\times P\times {S}_n}{P+{S}_n}\times 100\% $$

$$ Acc=\frac{TP+TN}{TP+TN+FP+FN}\times 100\% $$

where *TP*, *TN*, *FP*, and *FN* represent the number of true positive, true negative, false positive, and false negative cases, respectively.

## Additional file

Additional file 1:
**Information of selcted 500 RjRk.**
